# “I am there just to get on with it”: a qualitative study on the labour of the patient and public involvement workforce

**DOI:** 10.1186/s12961-024-01197-5

**Published:** 2024-09-02

**Authors:** Stan Papoulias, Louca-Mai Brady

**Affiliations:** 1 Health Service & Population Research, King’s College London, London, United Kingdom; 2 Centre for Public Health and Community Care, University of Hertfordshire, Hatfield, United Kingdom

**Keywords:** Patient and public involvement, Labour, PPI workforce, New public management, National Institute for Health and Care Research (NIHR)

## Abstract

**Background:**

Workers tasked with specific responsibilities around patient and public involvement (PPI) are now routinely part of the organizational landscape for applied health research in the United Kingdom. Even as the National Institute for Health and Care Research (NIHR) has had a pioneering role in developing a robust PPI infrastructure for publicly funded health research in the United Kingdom, considerable barriers remain to embedding substantive and sustainable public input in the design and delivery of research. Notably, researchers and clinicians report a tension between funders’ orientation towards deliverables and the resources and labour required to embed public involvement in research. These and other tensions require further investigation.

**Methods:**

This was a qualitative study with participatory elements. Using purposive and snowball sampling and attending to regional and institutional diversity, we conducted 21 semi-structured interviews with individuals holding NIHR-funded formal PPI roles across England. Interviews were analysed through reflexive thematic analysis with coding and framing presented and adjusted through two workshops with study participants.

**Results:**

We generated five overarching themes which signal a growing tension between expectations put on staff in PPI roles and the structural limitations of these roles: (i) the instability of support; (ii) the production of invisible labour; (iii) PPI work as more than a job; (iv) accountability without control; and (v) delivering change without changing.

**Conclusions:**

The NIHR PPI workforce has enabled considerable progress in embedding patient and public input in research activities. However, the role has led not to a resolution of the tension between performance management priorities and the labour of PPI, but rather to its displacement and – potentially – its intensification. We suggest that the expectation to “deliver” PPI hinges on a paradoxical demand to deliver a transformational intervention that is fundamentally divorced from any labour of transformation. We conclude that ongoing efforts to transform health research ecologies so as to better respond to the needs of patients will need to grapple with the force and consequences of this paradoxical demand.

## Introduction – the labour of PPI

The inclusion of patients, service users and members of the public in the design, delivery and governance of health research is increasingly embedded in policy internationally, as partnerships with the beneficiaries of health research are seen to increase its relevance, acceptability and implementability. In this context, a growing number of studies have sought to evaluate the impact of public participation on research, including identifying the barriers and facilitators of good practice [1–8]. Some of this inquiry has centred on power, control and agency. Attention has been drawn, for example, to the scarcity of user or community-led research and to the low status of experiential knowledge in the hierarchies of knowledge production guiding evidence-based medicine [9]. Such hierarchies, authors have argued, constrain the legitimacy that the experiential knowledge of patients can achieve within academic-led research [10], may block the possibility of equitable partnerships such as those envisioned in co-production [11] and may function as a pull back against more participatory or emancipatory models of research [12–14]. In this way, patient and public inclusion in research may become less likely to aim towards inclusion of public and patient-led priorities, acting instead as kind of a “handmaiden” to research, servicing and validating institutionally pre-defined research goals [15–17].

Research on how public participation-related activities function as a form of labour within a research ecosystem, however, is scarce [18]. In this paper, we examine the labour of embedding such participation, with the aim of understanding how such labour fits within the regimes of performance management underpinning current research systems. We argue that considering this “fit” is crucial for a broader understanding of the implementation of public participation and therefore its potential impact on research delivery. To this end, we present findings from a UK study of the labour of an emerging professional cadre: “patient and public involvement” leads, managers and co-ordinators (henceforth PPI, the term routinely used for public participation in the United Kingdom). We concentrate specifically on staff working on research partnerships and centres funded by the National Institute for Health and Care Research (NIHR). This focus on the NIHR is motivated by the organization’s status as the centralized research and development arm of the National Health Service (NHS), with an important role in shaping health research systems in the United Kingdom since 2006. NIHR explicitly installed PPI in research as a foundational part of its mission and is currently considered a global leader in the field [19]. We contend that exploring the labour of this radically under-investigated workforce is crucial for understanding what we see as the shifting tensions – outlined in later sections – that underpin the key policy priority of embedding patients as collaborators in applied health research. To contextualize our study, we first consider how the requirement for PPI in research relates to the overall policy rationale underpinning the organizational mission of the NIHR as the NHS’s research arm, then consider existing research on tensions identified in efforts to embed PPI in a health system governed through regimes of performance management and finally articulate the ways in which dedicated PPI workers’ responsibilities have been developed as a way to address these tensions.

### The NIHR as a site of “reformed managerialism”

The NIHR was founded in 2006 with the aim of centralizing and rationalizing NHS research and development activities. Its foundation instantiated the then Labour government’s efforts to strengthen and consolidate health research in the UK while also tackling some of the problems associated with the earlier introduction of new public management (NPM) principles in the governance of public services. NPM had been introduced in the UK public sector by Margaret Thatcher’s government, in line with similar trends in much of the Global North [20]. The aim was to curb what the Conservatives saw as saw as excesses in both public spending and professional autonomy. NPM consisted in management techniques adapted from the private sector: in the NHS this introduction was formalized via the 1990 National Health Service and Community Care Act, which created an internal market for services, with local authorities purchasing services from local health providers (NHS Trusts) [21]; top-down management control; an emphasis on cost-efficiency; a focus on targets and outputs over process; an intensification of metrics for performance management; and a positioning of patients and the public as consumers of health services with a right to choose [22, 23]. In the context of the NHS, cost-efficiency meant concentrating on services and on research which would have the greatest positive impact on population health while preventing research waste [24]. By the mid-1990s, however, considerable criticism had been directed towards this model, including concerns that NPM techniques resulted in silo-like operations and public sector fragmentation, which limited the capacity for collaboration between services essential for effective policy. Importantly, there was also a sense that an excessive managerialism had resulted in a disconnection of public services from public and civic aims, that is, from the values, voices and interests of the public [25, 26].

In this context, the emergence of the NIHR can be contextualized through the succeeding Labour government’s much publicized reformed managerialism, announced in their 1997 white paper “The New NHS: Modern, Dependable” [27]. Here, the reworking of NPM towards “network governance” meant that the silo-like effects of competition and marketization were to be attenuated through a turn to cross-sector partnerships and a renewed attention to quality standards and to patients’ voices [28]. It has been argued, however, that the new emphasis on partnerships did not undermine the dominance of performance management, while the investment in national standards for quality and safety resulted in an intensified metricization, with the result that this reform may have been more apparent than real, amounting to “NPM with a human face” [29–31]. Indeed, the NIHR can be seen as an exemplary instantiation of this model: as a centralized commissioner of research for the NHS, the NIHR put in place reporting mechanisms and performance indicators to ensure transparent and cost-efficient use of funds, with outputs and impact measured, managed and ranked [24]. At the same time, the founding document of the NIHR, Best Research for Best Health, articulates the redirection of such market-oriented principles towards a horizon of public good and patient benefit. The document firmly and explicitly positioned patients and the public as both primary beneficiaries of and important partners in the delivery of health research. People (patients) were to be placed “at the centre of a research system that focuses on quality, transparency and value for money” [32], a mission implemented through the installation of “structures and mechanisms to facilitate increased involvement of patients and the public in all stages of NHS Research & Development” [33]. This involvement would be supported by the advisory group INVOLVE, a key part of the new centralized health research system. INVOLVE, which had started life in 1996 as Consumers in NHS Research, funded by the Department of Health, testified to the Labour administration’s investment in championing “consumer” involvement in NHS research as a means of increasing research relevance [34]. The foundation of the NIHR then exemplified the beneficent alignment of NPM with public benefit, represented through the imaginary of a patient-centred NHS, performing accountability to the consumers/taxpayers through embedding PPI in all its activities. In this context, “public involvement” functioned as the lynchpin through which such alignment could be effected.

### PPI work and the “logic of deliverables”: a site of tension

Existing research on the challenges of embedding PPI has typically focussed on the experiences of academics tasked with doing so within university research processes. For example, Pollard and Evans, in a 2013 paper, argue that undertaking PPI work in mental health research can be arduous, emotionally taxing and time consuming, and as such, can be in tension with expectations for cost-efficient and streamlined delivery of research outputs [35]. Similarly, Papoulias and Callard found that the “logic of deliverables” governing research funding can militate against undertaking PPI or even constitute PPI as “out of sync” with research timelines [36]. While recent years have seen a deepening operationalization of PPI in the NIHR and beyond, there are indications that this process, rather than removing these tensions, may have recast them in a different form. For example, when PPI is itself set up as performance-based obligation, researchers, faced with the requirement to satisfy an increasing number of such obligations, may either engage in “surface-level spectacles” to impress the funder while eschewing the long-term commitment necessary for substantive and ongoing PPI, or altogether refuse to undertake PPI, relegating the responsibility to others [37, 38]. Such refusals may then contribute to a sharpening of workplace inequalities: insofar as PPI work is seen as “low priority” for more established academic staff, it can be unevenly distributed within research organizations, with precariously employed junior researchers and women typically assigned PPI responsibilities with the assumption that they possess the “soft skills” necessary for these roles [39].

Notably, the emergence of a dedicated PPI workforce is intended as a remedy for this tension by providing support, expertise and ways of negotiating the challenges associated with undertaking PPI responsibilities. In the NIHR, this workforce is part of a burgeoning infrastructure for public involvement which includes national standards, training programmes, payment guidelines, reporting frameworks and impact assessments [40–45]. By 2015, an INVOLVE review of PPI activities during the first 10 years of the NIHR attested to “a frenzy of involvement activity…across the system”, including more than 200 staff in PPI-related roles [40]. As NIHR expectations regarding PPI have become more extensive, responsibilities of PPI workers have proliferated, with INVOLVE organizing surveys and national workshops to identify their skills and support needs [41, 42]. In 2019, the NIHR mandated the inclusion of a “designated PPI lead” in all funding applications, listing an extensive and complex roster of responsibilities. These now included delivery and implementation of long-term institutional strategies and objectives, thus testifying to the assimilation of involvement activities within the roster of “performance-based obligations” within research delivery systems [43]. Notably however, this formalization of PPI responsibilities is ambiguous: the website states that the role “should be a budgeted and resourced team member” and that they should have “the relevant skills, experience and authority”, but it does not specify whether this should be a researcher with skills in undertaking PPI or indeed someone hired specifically for their skills in PPI, that is, a member of the PPI workforce. Equally, the specifications, skills and support needs, which have been brought together into a distinct role, have yet to crystallize into a distinct career trajectory.

Case studies and evaluations of PPI practice often reference the skills and expertise required in leading and managing PPI. Chief among them are relational and communication skills: PPI workers have been described as “brokers” who mediate and enable learning between research and lay spaces [44, 45]; skilled facilitators enabling inclusive practice [46–48]; “boundary spanners” navigating the complexities of bridging researchers with public contributors and undertaking community engagement through ongoing relational work [49]. While enumerating the skillset required for PPI work, some of these studies have identified a broader organizational devaluation of PPI workers: Brady and colleagues write of PPI roles as typically underfunded with poor job security, which undermines the continuity necessary for generating trust in PPI work [46], while Mathie and colleagues report that many PPI workers describe their work as “invisible”, a term which the authors relate to the sociological work on women’s labour (particularly housework and care labour) which is unpaid and rendered invisible insofar as it is naturalized as “care” [50]. Research on the neighbouring role of public engagement professionals in UK universities, which has been more extensive than that on PPI roles, can be instructive in fleshing out some of these points: public engagement professionals (PEPs) are tasked with mediating between academics and various publics in the service of a publicly accountable university. In a series of papers on the status of PEPs in university workplaces, Watermeyer and colleagues argue that, since public engagement labour is relegated to non-academic forms of expertise which lack recognition, PEPs’ efforts in boundary spanning do not confer prestige. This lack of prestige can, in effect, function as a “boundary block” obstructing PEPs’ work [51, 52]. Furthermore, like Mathie and Brady, Watermeyer and colleagues also argue that the relational and facilitative nature of engagement labour constitutes such labour as feminized and devalued, with PEPs also reporting that their work remains invisible to colleagues and institutional audit instruments alike [50, 53].

The present study seeks to explore further these suggestions that PPI labour, like that of public engagement professionals, lacks recognition and is constituted as invisible. However, we maintain that there are significant differences between the purpose and moral implications of involvement and engagement activities. PPI constitutes an amplification of the moral underpinnings of engagement policies: while public engagement seeks to showcase the public utility of academic research, public involvement aims to directly contribute to optimizing and personalizing healthcare provision by minimizing research waste, ensuring that treatments and services tap into the needs of patient groups, and delivering the vision of a patient-centred NHS. Therefore, even as PPI work may be peripheral to other auditable research activities, it is nevertheless central to the current rationale for publicly funded research ecosystems: by suturing performance management and efficiency metrics onto a discourse of public benefit, such work constitutes the moral underpinnings of performance management in health research systems. Therefore, an analysis of the labour of the dedicated PPI workforce is crucial for understanding how this suturing of performance management and “public benefit” works over the conjured figures of patients in need of benefit. This issue lies at the heart of our research study.

## Methods

Our interview study formed the first phase of a multi-method qualitative inquiry into the working practices of NIHR-funded PPI leads. While PPI lead posts are in evidence in most NIHR-funded research, we decided to focus on NIHR infrastructure funding specifically: these are 5-year grants absorbing a major tranche of NIHR funds (over £600 million annually in 2024). They function as “strategic investments” embodying the principles outlined in Best Research for Best Health: they are awarded to research organizations and NHS Trusts for the purposes of developing and consolidating capacious environments for early stage and applied clinical research, including building a research delivery workforce and embedding a regional infrastructure of partnerships with industry, the third sector and patients and communities [55]. We believe that understanding the experience of the PPI workforce funded by these grants may give better insights into NIHR’s ecosystem and priorities, since they are specifically set up to support the development of sustainable partnerships and embed the translational pipeline into clinical practice.

The study used purposive sampling with snowball elements. In 2020–2021, we mapped all 72 NIHR infrastructure grants, identified the PPI teams working in each of these using publicly available information (found on the NIHR website and the websites and PPI pages of every organization awarded infrastructure grants) and sent out invitation emails to all teams. Where applicable, we also sent invitations to mailing lists of PPI-lead national networks connected to these grants. Inclusion criteria were that potential participants should have oversight roles, and/or be tasked with cross-programme/centre responsibilities, meaning that their facilitative and strategy building roles should cover the entirety of activities funded by one (and sometimes more than one) NIHR infrastructure grant or centres including advisory roles over most or all research projects associated with the centre of grant, and that they had worked in this or a comparable environment for 2 years.

The individuals who showed interest received detailed information sheets. Once they agreed to participate, they were sent a consent form and a convenient interview time was agreed. We conducted 21 semi-structured interviews online, between March and June 2021, lasting 60–90 min. The interview topic guide was developed in part through a review of organizational documents outlining the role and through a consideration of existing research on the labour of PPI within health research environments. It focussed on how PPI workers fit within the organization relationship between the actual work undertaken and the way this work is represented to both the organization and the funder. Interview questions included how participants understand their role; how they fit in the organization; how their actual work relates to the job description; how their work is understood by both colleagues and public contributors; the relationship between the work they undertake and how this is represented in reports to funder and presentations; and what they find challenging about their work. Information about participants’ background and what brought them to their present role was also gathered. Audio files were checked, transcribed and the transcripts fully de-identified. All participants were given the opportunity to check transcripts and withdraw them at any point until December 2021. None withdrew.

We analysed the interviews using reflexive thematic analysis with participatory elements [54, 55]. Reflexive thematic analysis emphasizes the interpretative aspects of the analytical process, including the data “collection” process itself, which this approach recognizes as a generative act, where meaning is co-created between interviewer and participant and the discussion may be guided by the participant rather than strictly adhering to the topic guide [56]. We identified patterns of meaning through sustained and immersive engagement with the data. NVivo 12 was used for coding, while additional notes and memos on the Word documents themselves mitigated the over-fragmentation that might potentially limit NVivo as a tool for qualitative analysis. Once we had developed themes which gave a thorough interpretation of the data, we presented these to participants in two separate workshops to test for credibility and ensure that participants felt ownership of the process [57].

## Results

As the population from which the sample was taken is quite small, with some teams working across different infrastructure grants, confidentiality and anonymity were important concerns for participants. We therefore decided neither to collect nor to present extensive demographic information to preserve confidentiality and avoid deductive disclosure [58]. Out of our 21 participants 20 were women; there was some diversity in age, ethnicity and heritage, with a significant majority identifying as white (British or other European). Participants had diverse employment histories: many had come from other university or NHS posts, often in communications, programme management or human resources; a significant minority had come from the voluntary sector; and a small minority from the private sector. As there was no accredited qualification in PPI at the time this study was undertaken, participants had all learned their skills on their present or previous jobs. A total of 13 participants were on full-time contracts, although in several cases funding for these posts was finite and fragmented, often coming from different budgets.

In this paper we present five inter-related themes drawing on the conceptual architecture we outlined in the first half of this paper to explore how PPI workers navigate a research ecosystem of interlocking institutional spaces that is governed by “NPM with a human face”, while striving to align patients and the public with the imaginary of the patient-centred NHS that mobilizes the NIHR mission. These five themes are: (i) the instability of support; (ii) the production of invisible labour; (iii) PPI as moral imperative; (iv) accountability without control; and (v) delivering change without changing.

### “There to grease the cogs rather than be the cogs”: the instability of “support”

Infrastructure grants act as a hub for large numbers of studies, often in diverse health fields, most of which should, ideally, include PPI activities. Here, dedicated PPI staff typically fulfil a cross-cutting role: they are meant to oversee, provide training and advise on embedding PPI activities across the grant and, in so doing, support researchers in undertaking PPI. On paper, support towards the institution in the form of training, delivering strategy for and evaluating PPI is associated with more senior roles (designated manager or lead) whereas support towards so-called public contributors is the remit of more junior roles (designated co-ordinator or officer) and can include doing outreach, facilitating, attending to access needs and developing payment and compensation procedures. However, these distinctions rarely applied in practice: participants typically reported that their work did not neatly fit into these categories and that they often had to fulfil both roles regardless of their title. Some were the only person in the team specifically tasked with PPI, and so their “lead” or “manager” designation was more symbolic than actual:

*I have no person to manage, although sometimes I do get a little bit of admin support, but I don’t have any line management responsibility. It is really about managing my workload, working with people and managing the volunteers that I work with and administrating those groups and supporting them *(P11).

P11’s title was manager but, as they essentially worked alone, shuttling between junior and senior role responsibilities, they justified and made sense of their title by reframing their support work with public contributors as “management”. Furthermore, other participants reported that researchers often misunderstood PPI workers’ cross-cutting role and expected them to both advise on and deliver PPI activities themselves, even in the context of multiple projects, thus altogether releasing researchers of such responsibility.

*As a PPI lead, it is very difficult to define what your role is in different projects….and tasks … So, for example, I would imagine in [some cases] we are seen as the go-to if they have questions. [..] whereas, in [other cases], it is like, “Well, that’s your job because you’re the PPI lead” […] there is not a real understanding that PPI is everyone’s responsibility and that the theme leads are there to facilitate and to grease the cogs rather than be the cogs *(P20).

Furthermore, participants reported that the NIHR requirement for a PPI lead in all funding applications might in fact have facilitated this slippage. As already mentioned, the NIHR requirement does not differentiate between someone hired specifically to undertake PPI and a researcher tasked with PPI activities. The presence of a member of staff with a “PPI lead” title thus meant that PPI responsibilities in individual research studies could continue to accrue on that worker:

*The people who have been left with the burden of implementing [the NIHR specified PPI lead role] are almost exclusively people like me, though, because now researchers expect me to allow myself to be listed on their project as the PPI lead, and I actually wrote a document about what they can do for the PPI lead that more or less says, “Please don’t list me as your PPI lead. Please put aside funds to buy a PPI lead and I will train them, because there is only one me; I can’t be the PPI lead for everyone” *(P10).

This expectation that core members of staff with responsibilities for PPI would also be able to act as PPI leads for numerous research projects suggests that this role lacks firm organizational co-ordinates and boundaries. Here, the presence of a PPI workforce does not, in fact, constitute an appropriate allocation of PPI labour but rather testifies to a continuing institutional misapprehension of the nature of such labour particularly in terms of its duration, location and value.

### Conjuring PPI: the production of invisible labour

Participants consistently emphasized the invisibility of the kinds of labour, both administrative and relational, specific to public involvement as a process, confirming the findings of Mathie and colleagues [50]. This invisibility took different forms and had different justifications. Some argued that key aspects of their work, which are foundational to involvement, such as the process of relationship building, do not lend themselves to recognition as a performance indicator: “*There is absolutely no measure for that because how long is a piece of string” *(P11). In addition, relationship building necessitated a considerably greater time investment than was institutionally acceptable, and this was particularly evident when it came to outreach. Participants who did their work in community spaces told stories of uncomprehending line-managers, or annoyed colleagues who wondered where the PPI worker goes and what they do all day:

*There is very little understanding from colleagues about what I do on a day-to-day basis, and it has led to considerable conflict …. I would arrive at the office and then I would be disappearing quite promptly out into the community, because that is where I belong […] So, it is actually quite easy to become an absent person * (P3).

Once again, the NIHR requirement for designated PPI leads in funding applications, intended to raise the visibility of PPI work by formalizing it as costed labour, could instead further consolidate its invisibility:

*I am constantly shoved onto bids as 2% of my full-time equivalent and I think I worked out for a year that would be about 39 hours a year. For a researcher, popping the statistician down and all these different people on that bid, “Everyone is 2% and we need the money to run the trial, so 2% is fine”. And if I said to them, “Well, what do you think I would do in those 39 hours?” they wouldn’t have a clue, not a clue* (P17).

The 2% of a full-time allocation is accorded to the PPI worker because 2–5% is the time typically costed for leadership roles or for roles with a circumscribed remit (e.g. statisticians). However, this allocation, in making PPI workers’ labour visible either as oversight (what project leads do) or as methodological expertise (what statisticians do), ends up producing the wrong kind of visibility: the 39 h mentioned here might make sense when the role mainly involves chairing weekly meetings or delivering statistical models but are in no way sufficient for the intense and ongoing labour of trust-building and alignment between institutions and public contributors in PPI.

Indeed, such costings, by eliding the complexity and duration of involvement, may reinforce expectations that PPI can be simply conjured up at will and delivered on demand:

*A researcher will say to us, “I would really like you to help me to find some people with lived experience, run a focus group and then I’ll be away”. To them, that is the half-hour meeting to talk about this request, maybe 10 minutes to draft a tweet and an email to a charity that represents people with that condition […] the reality is it is astronomically more than that, because there is all this hidden back and forth. […] [researchers] expect to be able to hand over their protocol and then I will find them patients and those patients will be … representative and I will be able to talk to all of those patients and … write them up a report and …send it all back and they will be able to be like, “Thanks for the PPI”, and be on their merry way * (P13).

What P13 communicates in this story is the researcher’s failure to perceive the difference between PPI work and institutional norms for project delivery: the researcher who asks for “some people with lived experience” is not simply underestimating how long this process will take. Rather, involvement work is perceived as homologous to metricized and institutionally recognizable activities (for example, recruitment to trials or producing project reports) for which there already exist standard procedures. Here, the relational complexity and improvised dynamic of involvement is turned into a deliverable (“the PPI”) that can be produced through following an appropriate procedure. When PPI workers are expected to instantly deliver the right contributors to fit the project needs, PPI labour is essentially black boxed and in its place sits “the PPI”, a kind of magical object seemingly conjured out of nowhere.

Such invisibility, however, may also be purposefully produced by the PPI workers themselves. One participant spoke of this at length, when detailing how they worked behind the scenes to ensure public contributors have input into research documents:

*When we get a plain English summary from a researcher, we rewrite them completely. If the advisory group [see] … a really bad plain English summary, they are just going to go, “I don’t understand anything”. I might as well do the translation straight away so that they can actually review something they understand. [Researchers then] think, “Oh, [the public advisory group] are so good at writing” … and I am thinking, “Well, they don’t … write, they review, and they will say to me, ‘Maybe move this up there and that up there, and I don’t understand these’”, … They are great, don’t get me wrong, but they don’t write it. And it is the same with a lot of things. They think that [the group] are the ones that do it when it is actually the team *(P7).

Here, the invisibility of the PPI worker’s labour is purposefully wrought to create good will and lubricate collaboration. Several participants said that they chose to engage in such purposeful invisibility because they knew that resources were not available to train researchers in plain writing and public contributors in academic writing. PPI workers, in ghost-writing accessible texts, thus effect a shortcut in the institutional labour required to generate alignment between researchers and public contributors. However, this shortcut comes at a price: in effecting it, PPI workers may collude in conjuring “the PPI” – they may themselves make their own work disappear.

### “Not a 9 to 5”: PPI work as more than a job

Most participants reported that overtime working was common for themselves and their teammates, whether they were on a fractional or full-time contract. Overall, participants saw undertaking extra work as a necessary consequence of their commitment towards public contributors, a commitment which made it difficult to turn work down:

*Everyone loses if you say no: the public contributors aren’t involved in a meaningful way, the project won’t be as good because it doesn’t have meaningful PPI involvement *(P20).

While overwork was a common result of this commitment, some participants described such overwork as the feature that distinguished PPI work from what one commonly understands as a “job”, because, in this case, over-work was seen as freely chosen rather than externally imposed:

*It is me pushing myself or wanting to get things done because I started it and I think I would get less done if I worked less and that would bother me, but I don’t think it is a pressure necessarily from [line manager] or [the institution] or anyone to be like, “No, do more” *(P13).

Participants presented relationship building not only as the most time-consuming but also the most enjoyable aspect of PPI work. Community engagement was a key site for this and once again participants tended to represent this type of work as freely chosen:

*I did most of the work in my free time in the end because you have to go into communities and you spend a lot longer there. […] So, all of that kind of thing I was just doing in my spare time and I didn’t really notice at the time because I really enjoyed it * (P6).

Thus, time spent in relationship building was constituted as both work and not work. It did not lend itself to metricization via workplace time management and additionally, was not perceived by participants themselves as labour (“I didn’t really notice it at the time”). At the same time, out-of-hours work was rationalized as necessary for inclusivity, set up to enable collaboration with public contributors in so far as these do not have a contractual relationship to the employer:

*That is not a 9–5. That is a weekends and holidays sort of job, because our job is to reduce the barriers to involvement and some of those barriers are hours – 9–5 is a barrier for some people *(P17).

If working overtime allows PPI workers to reduce barriers and enable collaboration with those who are not employed by the institution, that same overtime work also serves to conceal the contractual nature of the PPI workers’ own labour, which now becomes absorbed into the moral requirements of PPI.

### “Caught in the middle”: accountability without control

Participants repeatedly emphasized that their ability to contribute to research delivery was stymied by their lack of control over specific projects and over broader institutional priority setting:

*… as a PPI lead we are not full member of staff, we are not responsible for choosing the research topics. We […] can only guide researchers who come to us and tell us what they are doing … we don’t have any power to define what the public involvement looks like in a research project *(P6).

Tasked with creating alignments and partnerships between the publics and institutions, participants argued that they did not have the power to make them “stick” because they are not “really” part of the team. However, even as PPI workers lacked the power to cement partnerships, any failure in the partnership could be ascribed to them, perceived as a failure of the PPI worker by both funder and public contributors:

*Often you have to hand over responsibility and the researcher [who] can let the panel down and … I feel like I have let the panel member down because … I am the one who said, “Oh yes, this person wants to talk to you”, and I find that really challenging, getting caught in the middle like that *(P21).

This pairing of accountability with lack of control became more pronounced in grant applications or reports to the funder:

*It is also quite frustrating in the sense that, just because I advise something, it doesn’t necessarily mean that it gets implemented or even included in the final grant. [even so] whatever the feedback is still reflects on us, not necessarily on the people who were making the wider decisions […] As PPI leads, we are still usually the ones that get the blame *(P10).

Several participants testified to this double frustration: having to witness their PPI plans being rewritten to fit the constraints (financial, pragmatic) of the funding application, they then often found themselves held accountable if the PPI plans fail to carry favour with the funder. PPI workers then become the site where institutional accountability to both its public partners and to the funder gathers – it is as though, while located outside most decision-making, they nevertheless become the attractors for the institution’s missing accountability, which they experience, in the words of P21, as “*being caught in the middle*” or, as another participant put it, as “*the worry you carry around*” (P16).

### “There to just get on with it”: delivering change without changing

Participants recognized that effective collaboration between research institutions and various publics requires fundamental institutional changes. Yet they also argued that while PPI workers are not themselves capable of effecting such change, there is nevertheless considerable institutional pressure to deliver on promises made in grant applications and build PPI strategies on this basis:

*So, there is that tension about […] pushing this agenda and encouraging people to do more [….] rather than just accepting the status quo. But actually, the reality is that it is very, very hard to get everybody in [grant name] to change what they do and I can’t make that happen, [senior PPI staff] can’t make that happen, nobody can. The whole systemic issue … But you have got, somehow in the strategy and what you say you are going to do, that tension between aspiration and reality *(P4).

This tension between aspiration and reality identified here could not be spelled out in reports for fear of reputational damage. In fact, the expectation to have delivered meaningful PPI, now routinely set up in NIHR applications, could itself militate against such change. For example, a frequently voiced concern was that PPI was being progressively under-resourced:

*I feel the bar is getting higher and higher and higher and expectations are higher and we have got no extra resource *(P16).

However, annual reports, the mechanism through which the doing of PPI is evidenced, made it difficult to be open about any such under-resourcing.

*We will allude to [the lack of resources]. So, we will say things like, “We punch above our weight”, but I am not sure that message gets home to the NIHR very clearly. It is not like the annual report is used to say, “Hey, you’re underfunding this systematically, but here’s all the good stuff we do”, because the annual report is, by essence, a process of saying how great you are, isn’t it? *(P3).

The inclusion of PPI as a “deliverable” meant that, in a competitive ecosystem, the pressure is on to report that PPI has always already been delivered. As another participant put it, “*no one is going to report the bad stuff*” (P17). Hence reporting, in setting up PPI as a deliverable, reinforced new zones of invisibility for PPI labour and made it harder to surface any under-resourcing for such labour. Furthermore, such reporting also played down any association between successful PPI and system transformation. Another participant described the resistance they encountered after arguing the organization should move away from “last-minute” PPI:

*I think it is really hard when […] these people are essentially paying your pay cheque, to then try to push back on certain things that I don’t think are truly PPI ….[A]s somebody who I felt my role was really to show best practice, for then [to be] seen as this difficult person for raising issues or pushing back rather than just getting things done, is really hard [….] I get the impression, at least within the [organization] … that I am not there to really point out any of the issues. I am there just to get on with it *(P14).

This opposition between pointing out the issues and “getting on with it” is telling. It names a contradiction at the heart of PPI labour: here, the very act of pushing back – in this case asking for a commitment to more meaningful and ongoing PPI – can be perceived as going against the PPI worker’s responsibilities, insofar as it delays and undoes team expectations for getting things done, for delivering PPI. Here, then, we find an exemplary instance of the incommensurability between the temporal demands of research and those of meaningful PPI practice.

## Discussion

How do the five themes we have presented help open out how policies around public participation are put into practice—as well as the contradictions that this practice navigates – in health systems organized by the rhetorical suturing of performance management onto public benefit? We have argued that the development of a dedicated workforce represents an attempt to “repair” the tension experienced by researchers between the administrative, facilitative and emotional work of PPI and the kinds of deliverables that the institution requires them to prioritize. We argue that our findings indicate that insofar as PPI workers’ role then becomes one of “delivering” PPI, this tension is reproduced and at times intensified within their work. This is because, as actors in the health research ecosystem, PPI staff are tethered to the very regimes of performance management, which give rise to an institutional misapprehension of the actual labour associated with delivering PPI.

This misapprehension surfaces in the instruments through which the funder costs, measures and generates accountability for PPI – namely, the requirement for a costed PPI lead and the mandatory inclusion of a PPI section in applications and regular reports to funder. The NIHR requirement for a costed PPI lead, intended to legitimize the undertaking of PPI as an integral part of a research team’s responsibilities, may instead continue to position the PPI worker as a site for the research team’s wholesale outsourcing of responsibility for PPI, since this responsibility, while in tension with other institutional priorities, cannot nevertheless be refused by the team. Furthermore, the use of titles such as lead, manager or co-ordinator not only signal an orderly distinction between junior and senior roles, which often does not apply in practice, but also reframes the extra-institutional work of PPI (the forging of relationships and administrative support with public contributors), through the intra-institutional functions of performance/project management. This reframing elides an important difference between the two: public and patient partners, for the most part, do not have a formal contractual relationship with the institution and are not subject to performance management in the way that contracted researchers and healthcare professionals are. Indeed, framing the relationship between PPI workers and public contributors through the language of “management” fundamentally misrecognizes the kinds of relationalities produced in the interactions between PPI workers and public contributors and elides the externality of PPI to the “logic of deliverables” [36].

The inclusion of a detailed PPI section in grant applications and annual reports to funder further consolidates this misapprehension by also representing public involvement as if it is already enrolled within organizational normative procedures and therefore compels those in receipt of funding to evidence such delivery through annual reports [37]. This demand puts PPI workers under increasing pressure, since their function is to essentially present PPI objectives as not only achievable but already achieved, thus essentially bracketing out the process of organizational transformation which is a necessary prerequisite to establishing enduring partnerships with patients and the public. This bracketing out is at work in the organizational expectation to “just get on with it”, which structures the labour of delivering PPI in NIHR-funded research. Here, the demand to just get on, to do the work one is paid to do, forecloses the possibility of engaging with the structural obstacles that militate against that work being done. To the extent that both role designation and reporting expectations function to conceal the disjuncture that the establishment of public partnerships represents for regimes of performance management, they generate new invisibilities for PPI workers. These invisibilities radically constrain how such labour can be adequately undertaken, recognized and resourced.

In suggesting that much of the labour of staff in public involvement roles is institutionally invisible, and that organizational structures may obstruct or block their efforts, we concur with the arguments made by Watermeyer, Mathie and colleagues about the position of staff in public engagement and public involvement roles, respectively. However, our account diverges from theirs in our interpretation of how and why this labour is experienced as invisible and how that invisibility could be remedied. Mathie and colleagues in particular attribute this invisibility to a lack of parity and an institutional devaluation of what are perceived as “soft skills” – facilitation and relationship building in particular [50]. They therefore seek to raise PPI work to visibility by emphasizing the complexity of PPI activities and by calling for a ring-fencing of resources and a development of infrastructures capable of sustaining such work. While we concur that the invisibility of PPI labour is connected to its devaluation within research institutions, we also suggest that, in addition, this invisibility is a symptom of a radical misalignment between regimes of performance management and the establishment of sustainable public partnerships. Establishing such partnerships requires, as a number of researchers have demonstrated [18, 59, 60], considerable institutional transformation, yet those tasked with delivering PPI are not only not in a position to effect such transformation, they are also compelled to conceal its absence.

Recognizing and addressing the misalignment between regimes of performance management and the establishment of sustainable public partnerships becomes particularly pressing given the increasing recognition, in many countries, that public participation in health research and intervention development is an important step to effectively identifying and addressing health inequalities [19, 61, 62]. Calls for widening participation, for the inclusion of under-served populations and for co-designing and co-producing health research, which have been gathering force in the last 20 years, have gained renewed urgency in the wake of the coronavirus disease 2019 (COVID-19) pandemic [63–67]. In the United Kingdom, Best Research for Best Health: The Next Chapter, published by the NIHR in 2021 to define the direction and priorities for NHS Research for the coming decade, exemplifies this urgency. The document asserts that a radical broadening of the scope of PPI (now renamed “public partnerships”) is essential for combatting health inequalities: it explicitly amplifies the ambitions of its 2006 predecessor by setting up as a key objective “close and equitable partnerships with communities and groups, including those who have previously not had a voice in research” [68]. Here, as in other comparable policy documents, emphasis on extending partnerships to so-called underserved communities rests on the assumption that, to some degree at least, PPI has already become the norm for undertaking research. This assumption, we argue, closes down in advance any engagement with the tensions we have been discussing in this paper, and in so doing risks exacerbating them. The document does recognize that for such inclusive partnerships to be established institutions must “work differently, taking research closer to people [..] and building relationships of trust over time” – though, we would suggest, it is far from clear how ready or able institutions are really to take on what working differently might mean.

Our study engages with and emphasizes this need to “work differently” while also arguing that the demands and expectations set up through regimes of performance management and their “logic of deliverables” are not favourable to an opening of a space in which “working differently” could be explored. In health research systems organized through these regimes, “working differently” is constrained by the application of the very templates, instruments and techniques which constitute and manage “business as usual”. Any ongoing effort to transform health research systems so as better to respond to growing health inequalities, our study implies, needs to combat, both materially and procedurally, the ease with which the disjuncture between embedding public partnerships and normative ways of undertaking research comes to disappear.

## Limitations

We focus on the labour of the PPI workforce and their negotiation of performance management regimes, which means that we have not discussed relationships between PPI staff and public contributors nor presented examples of good practice. While these are important domains for study if we are to understand the labour of the PPI workforce, they lie outside the scope of this article. Furthermore, our focus on the UK health research system means that our conclusions may have limited generalizability. However, both the consolidation of NPM principles in public sector institutions and the turn to public and patient participation in the design and delivery of health research are shared developments across countries in the Global North in the last 40 years. Therefore, the tensions we discuss are likely to also manifest in health systems outside the United Kingdom, even as they may take somewhat different forms, given differences in how research and grants are costed, and roles structured. Finally, this project has elements of “insider” research since both authors, while working primarily as researchers, have also had experience of embedding PPI in research studies and programmes. Insider research has specific strengths, which include familiarity with the field and a sense of shared identity with participants which may enhance trust, facilitate disclosure and generate rich data. In common with other insider research endeavours, we have sought to reflexively navigate risks of bias and of interpretative blind spots resulting from over-familiarity with the domain under research [69] by discussing our findings and interpretations with “non-insider” colleagues while writing up this research.

## Conclusions

Our qualitative study is one of the first to investigate how the UK PPI workforce is negotiating the current health research landscape. In doing so, we have focused on the UK’s NIHR since this institution embodied the redirection of performance management regimes towards public benefit by means of public participation. If PPI is set up as both the means of enabling this redirection and an outcome of its success, then the PPI workforce, the professional cadre evolving to support PPI, becomes, we argue, the site where the tensions of attempting this alignment are most keenly experienced.

We suggest that, while such alignment would demand a wholesale transformation of organizational norms, the regimes of performance management underpinning research ecologies may also work to foreclose such transformation, thus hollowing out the promise of patient-centred research policies and systems. Recognizing and attending to this foreclosure is urgent, especially given the current policy emphasis in many countries on broadening the scope, ambition and inclusivity of public participation as a means of increasing the reach, relevance and potential positive impact of health research.

## Data Availability

The data that support the findings of this study are available on request from the corresponding author.
